# From Insult to Injury: Exploring the Associations Between Severe Malnutrition in Childhood, Rehabilitation Weight Gain and Adult Adiposity in a Prospective Cohort Study

**DOI:** 10.1111/mcn.70101

**Published:** 2025-09-29

**Authors:** Debbie S. Thompson, Kimberley McKenzie, Asha Badaloo, Charles Opondo, Jonathan Wells, Mubarek Abera, Amir Kirolos, Albert Koulman, Marko Kerac, Michael S. Boyne, the CHANGE Study Collaborators

**Affiliations:** 1 Caribbean Institute for Health Research, The University of the West Indies, Kingston, Jamaica; 2 Department of Medical Statistics, Faculty of Epidemiology & Population Health, London School of Hygiene & Tropical Medicine, London, UK; 3 Childhood Nutrition Research Centre, Population, Policy and Practice Research and Teaching Department, University College London (UCL) Great Ormond Street Institute of Child Health, London, UK; 4 Department of Psychiatry, Faculty of Medical Science, Jimma University, Jimma, Ethiopia; 5 Department of Women and Children’s Health, Institute of Life Course and Medical Sciences, University of Liverpool, Liverpool, UK; 6 Core Metabolomics and Lipidomics Laboratory, Metabolic Research Laboratories, Institute of Metabolic Science, University of Cambridge, Cambridge, UK; 7 Department of Population Health, Faculty of Epidemiology and Population Health, London School of Hygiene and Tropical Medicine, London, UK; 8 Centre for Maternal, Adolescent & Reproductive Child Health (MARCH), London School of Hygiene & Tropical Medicine, London, UK; 9 Department of Medicine, The University of the West Indies, Kingston, Jamaica

**Keywords:** adiposity, cardiometabolic disease, malnutrition, noncommunicable disease, risk factors, weight gain

## Abstract

The relationships between severe malnutrition (SM), rehabilitation weight gain, and cardiometabolic risk in adult survivors have not been fully elucidated. We utilised a previously collected data set to explore these associations in a cohort of adults who were hospitalised for SM as children from 1963 to 1995. We studied 278 adult SM survivors: 60% male; median age (IQR) 26.5(11.3) years; mean BMI 23.6(5.2) kg/m^2^). Children’s minimum weight-for-age z scores after hospitalisation (minWAZ) were analysed against adiposity as adults in sex-disaggregated regression models. Higher minWAZ was associated with greater adult waist circumference (mean difference:1.8 cm, 95%CI 0.7, 2.9, *p* = 0.001), fat mass (difference:2.4 kg, 95%CI 0.17,1.06, *p* = 0.007) and android fat mass (difference:0.19 kg, 95%CI 0.09, 0.29, *p* < 0.001) in bivariate analyses. Approximately 13% of the effect of minWAZ on adult fat mass was mediated by rehabilitation weight gain in g/kg/day (Sobel’s *p* = 0.053). In male and not female adult survivors, rehabilitation weight gain > 12.9 g/kg/day was associated with greater adult fat mass (difference:5 kg, 95%CI 2, 9, *p* = 0.006) and android fat (difference:0.5 kg, 95%CI 0.1, 0.8, *p* = 0.006). Female sex was the strongest predictor of adult fat mass (difference:12.7 kg, 95%CI 9.6, 15.7, *p* < 0.001) and android fat mass (difference:0.9 kg, 95%CI 0.6, 1.2 *p* < 0.001) and adult age the strongest predictor of adult waist circumference (difference:0.67 cm, 95%CI 0.39, 0.94, *p* < 0.001). Faster rehabilitation weight gain as an independent, causal risk factor for adiposity in male SM survivors requires further exploration and more modest weight gain targets may contribute to reducing their risk of adult cardiometabolic disease.

## 1 Introduction

There is increasing evidence that severe malnutrition (SM) (Kerac et al. 2020) in early life is associated with later life cardiometabolic non-communicable disease (NCD) (Grey et al. 2021); a problem that is becoming increasingly relevant as many children are still exposed to SM, but more children survive the acute episode. Although several studies suggest associations between early postnatal exposure to SM and adult NCD risk (Lelijveld et al. 2023; Tennant et al. 2014; Thompson et al. 2020; Thompson et al. 2023), the mechanisms remain unclear. The focus of nutritional rehabilitation during SM is, quite rightly, the prevention of death. Traditionally, rapid restoration of normal anthropometry is seen as the best way to achieve this aim. However, rapid weight gain in infancy and early childhood is thought to be a predictor of increased risk of obesity in later life, including the developmental programming of high blood pressure (Lule et al. 2018) and risk of type 2 diabetes (Olaiya et al. 2020). This is especially so in situations where the weight gain is preceded by a period of undernutrition (Nobili et al. 2008). Similarly, we demonstrated that in adults who were hospitalised for SM in early childhood, faster weight gain during nutritional rehabilitation was associated with greater liver fat (Thompson et al. 2022) and that rehabilitation weight gain exceeding 12.9 g/kg/day was positively associated with adult BMI, waist circumference, fat mass and android fat (Thompson et al. 2023).

Additionally, *in utero* undernutrition, evidenced by low birth weight, is an important determinant of adult cardiometabolic risk with substantial evidence supporting an association between low birth weight and subsequent elevated blood pressure, impaired glucose tolerance and insulin resistance (Eriksson et al. 2000; Levitt et al. 2000; Li et al. 2001). Along with low birth weight and the degree of underweight, sex might play role in the long-term outcomes of early childhood SM. Since boys grow quicker *in utero*, albeit with less placental growth, their risk of intrauterine growth restriction and thus consequent postnatal undernutrition is increased (Eriksson et al. 2010), and it follows that male sex might influence the risk of severe malnutrition and its sequelae. Furthermore, other factors such as the age at SM diagnosis and the presence of nutritional oedema could influence the development of cardiometabolic NCD risk in adult survivors of SM; each constituting a potential influence very early in the life course. However, the relative impacts are still unknown and could possibly be cumulative.

**Figure 1:**
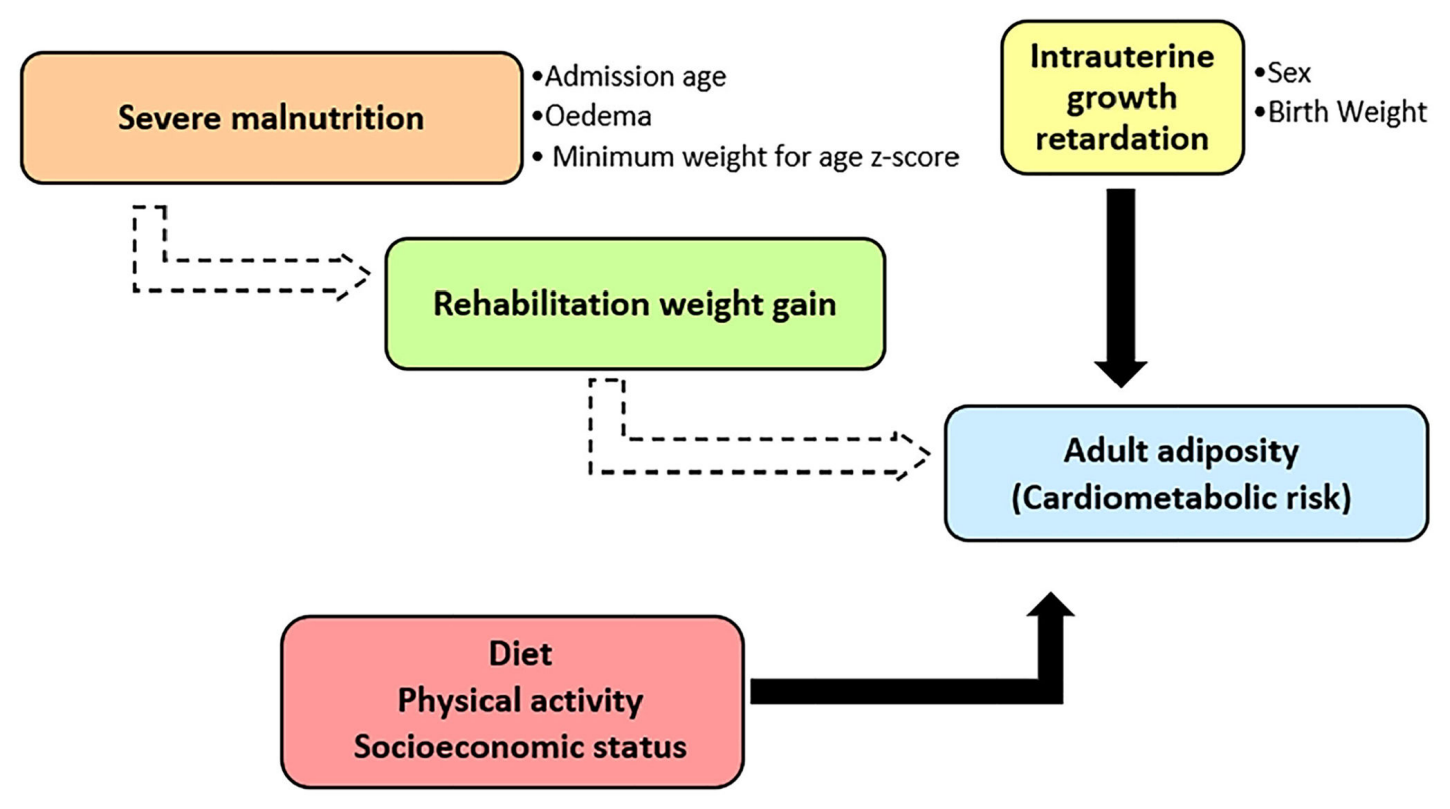
Conceptual framework depicting the variables that may potentially influence the relationship between severe malnutrition in early life and adiposity and cardiometabolic risk in adult survivors of severe malnutrition. Solid arrows represent established associations and dashed arrows represent hypothesised associations.

This study builds on our previous work (Thompson et al. 2023), and we utilised the previously collected data set to specifically to examine factors that could influence our earlier findings of associations between early childhood rehabilitation weight gain and adult adiposity (Figure 1). We hypothesise that sex, the presence of oedema and the rate of weight gain during treatment may influence the association between undernutrition in childhood and adult cardiometabolic NCD risk.

## 2 Methods

### Setting

The LION (Long-term Implications of Nutrition) Cohort was retrospectively assembled from individuals who were admitted to the Tropical Metabolism Research Unit (TMRU) ward at the University Hospital of the West Indies between the years 1963–1993 with a diagnosis of SM. At that time, National Centre for Health Statistics (NCHS) growth standards were utilised, and the focus was on inpatient management of all affected children; unlike the community-based management approaches in current use (WHO 1999b),(WHO 2023). Members of the cohort were followed up and extensively characterised as adults between 2008 and 2012 in the Jamaica Marasmus and Kwashiorkor Adult Survivors (JAMAKAS) Study (Francis-Emmanuel et al. 2014; Tennant et al. 2014; Thompson et al. 2020; Thompson et al. 2022) (Supporting Fig. 1). The Child malnutrition & Adult NCD: Generating Evidence on mechanistic links to inform future policy/practice (CHANGE) study involves secondary analysis of the data collected from these adults for whom childhood hospital admission records were available (Kerac et al. 2022). The Mona Campus Research Ethics Committee of the University of the West Indies (CREC-MN. 204 20/21) and the London School for Hygiene and Tropical Medicine (LSHTM Ethics Ref: 27722) approved the secondary analysis. All participants provided written informed consent.

During the original hospitalisation for SM, treatment began with an acute “stabilisation” phase in which a milk-based diet of 80–100 kcal/kg/day was fed to provide energy for weight maintenance, while infections and fluid imbalance were treated (WHO 1999a). Since the 1960s, metabolism research has informed development of treatment guidelines in the TMRU (Waterlow et al. 1992), which led to a gradual standardisation of the approach by about the mid-1970s (Picou et al. 1975) and helped to shape World Health Organisation (WHO) treatment guidelines of 1981 (WHO 1981). After stabilisation, resolution of oedema and improved appetite and affect, a brief “transition” phase would follow, allowing for a gradual increase in the amount of therapeutic food offered to the child. Thereafter the “rehabilitation” phase of treatment would begin where children were fed increasing amounts of energy and protein-enriched feeds, driven by the child’s appetite, to attain a body weight similar to WHO (1981) guidelines (WHO 1981). The final phase of treatment, referred to as “recovery,” involved weaning the child to an age-appropriate mixed diet before discharge from hospital. Children were discharged once they attained a weight-for-height ≥ 90% using NCHS reference values (WHO 1981) and were followed up as outpatients for up to 2 years post-hospitalisation. During this period, they were switched to a home-based diet which typically at the time in Jamaica consisted of animal protein and carbohydrates sources (e.g., potatoes, yams, pumpkin) taken from the family pot.

### Study Data

In this study, we utilised a previously collected data set (originally compiled for an earlier study) for a new analysis aimed at exploring additional insights. Childhood data were abstracted from hospital records and include birth weight, admission weight and length/height, minimum weight during admission (after loss of oedema), delta WAZ (ΔWAZ) taken from the minimum WAZ to maximum WAZ, weight and length/height measured throughout nutritional rehabilitation.

We utilised the following measures of rehabilitation weight gain: change in weight-for-age z-score (WAZ/day) using WHO Multicentre Growth Reference Study Group 2006 growth standards, as well as g/kg of body weight/day and weight in grams/day, which are all common measures of growth in malnutrition programmes.

Adult data include detailed medical and drug history, anthropometry, blood pressure, body composition (assessed using bioelectrical impedance analysis (BIA) and dual-energy X-ray absorptiometry (DEXA) (Lunar Prodigy, GE Health Care, USA), fasting glucose and fasting insulin with methods described elsewhere (D. S. Thompson et al. 2022).

In this secondary analysis, the main exposure variable was minimum WAZ (minWAZ), i.e., WAZ at the time of minimum weight (a proxy for malnutrition severity). Covariates included sex, age at minimum WAZ (minWAZage), oedema (present = 1, absent = 0) and rehabilitation weight gain and these were evaluated as mediators, effect modifiers and confounders. Rehabilitation weight gain was expressed as change in WAZ/day (ΔWAZ/day) using the formula (maxWAZ - min WAZ)/number of days and g/kg/day during nutritional rehabilitation and calculated using the formula: (weight gain in grams/number of days)/mean (max and minimum weight) in kilograms = weight gain in g/kg/day.

Participants were grouped into quintiles 1–5 (Q1–Q5) representing the slowest (Q1) and fastest (Q5) rates of each measure of rehabilitation weight gain. The range of outcome variables included waist circumference, total and percent fat mass, fat mass index and total and percent android fat mass.

### Statistical analyses

Unadjusted analyses were conducted to explore the associations between birth weight, minWAZ, minWAZage, presence of oedema, rehabilitation weight gain and measures of adult adiposity. Since adult men and women tend to have distinct body composition, measures of adult adiposity were regressed against minWAZ, in sex-disaggregated models adjusted for oedema, quintiles of rehabilitation weight gain and adult age. Additionally, all outcome measures of body composition were adjusted for adult height in separate regression models. Models adjusting for adult BMI were conducted but were not included in the final analyses as BMI was considered to be highly collinear with the outcome measures of adult adiposity.

Linear regression analyses were conducted to test for confounding variables; the Baron and Kenny approach (Baron and Kenny 1986) was used to test for mediation and the Ratio of Indirect to Total Effect (RIT) was used to quantify the proportion of the total effect on the dependent variable that is mediated by the mediator variable. An informal forward variable selection approach was used to identify predictors of adult adiposity using a *p* value ≤ 0.05 as the criterion for inclusion. Based on the conceptual framework of the study, seven (7) independent variables were identified for this analysis: documented birth weight, sex, oedema, minWAZ, minWAZ age, rehabilitation weight gain and adult age. Stata version 16.0 (Stata Corp LLC, College Station, Texas, USA) was used to conduct the statistical analyses and *p*-values ≤ 0.05 were considered statistically significant.

## 3 Results

### 3.1 Cohort Characteristics

Of the 1,366 children admitted between 1963 and 1993, 47 died during treatment for malnutrition and the remaining 1289 were discharged alive (mortality of 3.6%). The JAMAKAS Study (2008-2012) traced 729 adult survivors and enrolled 316 of them. Supporting Figure 1 summarises the recruitment of the adult survivors of SM.

Of the 316 adult SM survivors previously studied, the CHANGE study identified 278 for whom childhood admission records were available for secondary data analysis. We included data from these adults (60% male, median age (IQR) 26.5 (11.3) years, mean BMI (SD) 23.6 (5.2) kg/m^2^) in our analysis. The mean birth weight of the participants was 2.82 ± 0.78 kg (*n* = 152), mean admission age for SM was 10.9 months and 65% had oedematous malnutrition (Table 1). At admission, 88% of the children were stunted (HAZ < −2), 77% and 87% were wasted (using WHZ < −2 and MUAC < 12.5 cm respectively). Compared to girls, boys were more stunted (HAZ), more wasted (WHZ) and more underweight (WAZ) at the time of admission to hospital (Table 1). Rates of weight gain in g/kg/day were grouped into quintiles as follows Q1: 0.3–6.9 g/kg/day, Q2: 7.0–8.8 g/kg/day, Q3: 8.9–10.6 g/kg/day, Q4: 10.7–12.8 g/kg/day, Q5: 12.9–27.4 g/kg/day. Boys had similar rates of weight gain to girls during rehabilitation and were more underweight at the time of discharge from hospital (Table 1).

As adults, men were taller with greater lean mass and a higher lean mass index compared to women. Women had greater BMI, waist circumference, fat mass, fat mass index, and android fat mass compared to men (Table 1).

### 3.2 Associations of Birth Weight With Rehabilitation Weight Gain and Outcomes

In bivariate analyses, birth weight was not associated with any measure of rehabilitation weight gain i.e., ΔWAZ/day (*p* = 0.53), g/kg/day (*p* = 0.20), g/day (*p* = 0.34) or adult adiposity i.e., adult waist circumference (*p* = 0.7), fat mass (*p* = 0.85) or android fat (*p* = 0.65). However, birth weight was associated with greater adult lean mass (difference 3.22, 95% CI 1.2, 5.2, *p* = 0.002) (Supporting Information S1: Table 1).

### 3.3 Associations of Age and Weight at Admission With Rehabilitation Weight Gain and Outcomes

Age at the time of lowest weight-for-age z scores (minWAZage) was positively associated with rehabilitation weight gain in ΔWAZ (difference 0.0007, 95% CI 0.0001, 0.0012, *p* < 0.023) but was not associated with any measure of adult adiposity or lean mass (*p* > 0.35) (Supporting Information S1: Table 1).

Children who were least underweight initially (highest minWAZ) had faster rehabilitation weight gain when expressed in g/day (difference 2.7 g/day, 95% CI 0.5, 4.8, *p* = 0.016) but conversely, when weight gain was expressed as g/kg/day, children who were least underweight initially (highest minWAZ) had slower rehabilitation weight gain (difference −0.28 g/kg/day, 95% CI −0.40, −0.16, *p* < 0.001).

Children who were least underweight initially had higher later-life waist circumference (difference 1.78 cm, 95% CI 0.70, 2.90, *p* = 0.001), fat mass (difference 2.4 kg, 95% CI 1.4, 3.5, *p* < 0.001), fat mass index (difference 0.86 kg/m^2^, 95% CI 0.44, 1.3, *p* < 0.001) and android fat mass (difference 0.19 kg, 95% CI 0.09, 0.29, *p* < 0.001) than those who were most underweight initially (Supporting Information S1: Table 1).

### 3.4 Associations of Oedema With Adult Outcomes

Childhood oedematous malnutrition was associated with higher adult waist circumference (difference 5.7 cm, 95% CI 2.6, 8.8, *p* < 0.001), fat mass (difference 4.2 kg, 95% CI 0.97, 7.50, *p* = 0.01), fat mass index (difference 1.5 kg/m^2^, 95% CI 0.29, 2.80, *p* = 0.016), android fat (difference 0.33 kg, 95% CI 0.04, 0.62, *p* = 0.025), and lean mass (difference 3.6 kg, 95% CI 1.0, 6.1, *p* = 0.005) (Supporting Information S1: Table 1).

### 3.5 Associations Between Rehabilitation Weight Gain and Adult Outcomes

Measures of rehabilitation weight gain were variably associated with measures of adult adiposity, viz., rehabilitation weight gain expressed as change in WAZ/day (ΔWAZ/day) was associated with waist circumference (difference 1.2 cm, 95% CI 0.13, 2.2, *p* = 0.03) and fat mass (difference 1 kg, 95% CI 0.09, 2.3, *p* = 0.03) (Supporting Table 1) while rehabilitation weight gain expressed as g/kg/day was neither associated with adult adiposity nor lean mass (Supporting Information S1: Table 1). Finally, female sex was associated with greater adult BMI, waist circumference, fat mass, fat mass index, android fat mass and % android fat mass (Supporting Information S1: Table 1).

### 3.6 Sex-Disaggregated Regression Models of Adult Measures of Adiposity Against Minwaz

Three measures of adiposity, waist circumference (cm), total fat mass (kg) and android fat mass (kg), were selected as markers of adult cardiometabolic risk. BMI was excluded as it has documented limitations as a measure of adiposity (Rothman 2008), including the fact that it does not take into account the person’s body fat versus lean mass. Sex-disaggregated regression analyses of adult adiposity on min-WAZ in which models were adjusted for oedema, rehabilitation weight gain and adult age (Table 2) were conducted. When the sexes were combined, all the associations between minWAZ and the measures of adult adiposity are significant, but when the group was separated by sex, minWAZ was only associated with waist circumference among females in the model adjusted for age (Table 2). While regression models in which quintile scores of 1–5 of g/kg/day are presented in Table 2; it is to be noted that regression models adjusted for rehabilitation weight gain quintiles in ΔWAZ/day led to similar conclusions.

### 3.7 Predictors of Adult Adiposity in Adult Survivors of SM

Seven potential predictors of adult adiposity (birth weight, minWAZ, minWAZage, oedema, rehabilitation weight gain, sex and adult age) were used in informal forward selection regression analyses. The output, which featured only those variables that were significant, suggests that female sex most strongly predicted adult fat mass (difference 12.7 kg, 95% CI 9.6, 15.7, *p* < 0.001) and android fat mass (difference 0.91 kg, 95% CI 0.63, 1.2, *p* < 0.001), and adult age was the strongest predictor of adult waist circumference (difference 0.67 cm, 95% CI 0.39, 0.94, *p* < 0.001) (Supporting Information S1: Table 2).

**Figure 2:**
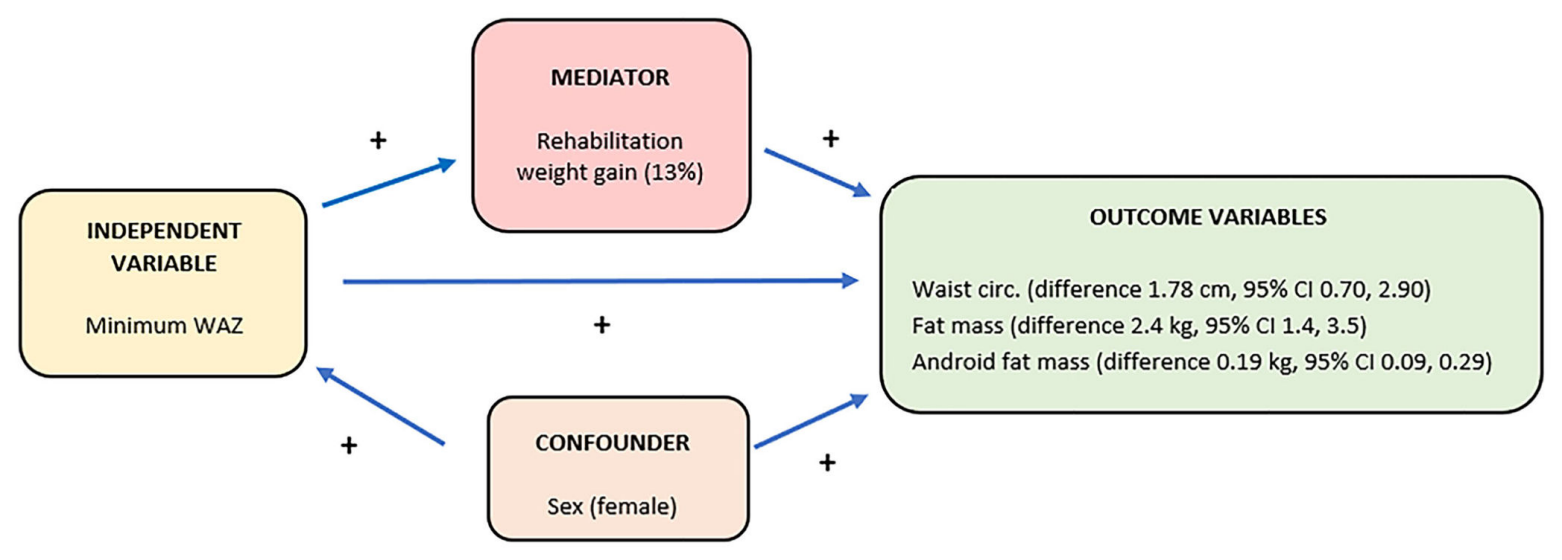
Summary of the variables demonstrated to contribute to the associations between severe malnutrition and adult adiposity. + positive association.

In unadjusted sex-aggregated analyses, minWAZ was associated with waist circumference, adult fat mass, and adult android fat mass. Using the Baron and Kenny approach, there was strong evidence of correlation between minWAZ and adult fat mass (*p* < 0.001) (Step 1), minWAZ and rehabilitation weight gain in g/kg/day (*p* < *0.001*) (Step 2) and rehabilitation weight gain (g/kg/day) and adult fat mass (*p* = 0.036) (step 3). Sobel’s test showed evidence of partial mediation by rehabilitation weight gain in g/kg/day (*p* = 0.053) in the association between childhood minWAZ and adult fat mass. The RIT (ratio of independent effect: total effect) was 0.13, i.e., roughly 13% of the effect of minWAZ on adult fat mass is mediated by rehabilitation weight gain in g/kg/day. (Figure 2).

In linear regression analysis conducted to test for confounding variables, female sex did not influence the relationship between minWAZ and waist circumference, but it confounded the relationships between minWAZ and both adult fat mass and android fat mass, increasing the coefficients by 180% and 160% respectively. Additionally, oedema did not influence the relationships between minWAZ and adult fat mass.

**Figure 3:**
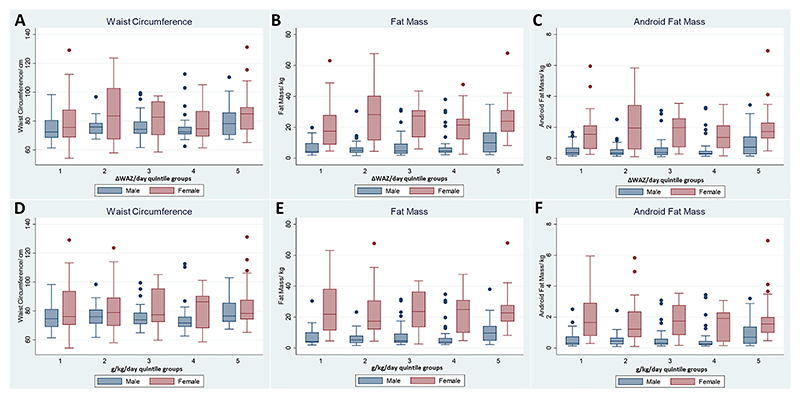
Adult age and minimum WAZ-adjusted linear regression analyses of the associations between rehabilitation weight gain and measures of adult adiposity in male and female adult SM survivors. Results of quintile group 1 vs 5 (difference, 95% CI, *p* value) (A–C): **ΔWAZ/day** - waist circumference (men: 6.1 cm, 95% CI [2, 10], *p* = 0.004, women: 7.7 cm, 95% CI [−0.3, 15], *p* = 0.058), fat mass (men: 4.3 kg, 95% CI [0.7, 8], *p* = 0.019, women: 4.3, 95% CI [−3.0, 12], *p* = 0.245) and android fat mass (men: 0.4 kg, 95% CI [0.07, 0.7], *p* = 0.018, women: 0.35 kg, 95% CI [−0.3, 1.0], *p* = 0.312); (D–F): **g/kg/day**-waist circumference (men: 6.6 cm, 95% CI [2, 11], *p* = 0.002, women: 7.2 cm, 95% CI [−1, 15], *p* = 0.086), fat mass (men: 5 kg, 95% CI [2, 9], *p* = 0.006, women: 4.2 kg, 95% CI [−3, 12], *p* = 0.267) and android fat mass (men: 0.48 kg, 95% CI [0.1, 0.8], *p* = 0.006, women: 0.28 kg, 95% CI [−0.4, 1.0], *p* = 0.436).

In sex-disaggregated analyses, rehabilitation weight gain that exceeded the threshold of 12.9 g/kg/day (Q5) was associated with waist circumference (6.6 cm, 95% CI 2, 11, *p* = 0.002) (Figure 3E), adult fat mass (difference = 5.1 kg, 95% CI 2.0, 9.0, *p* = 0.006) (Figure 3E) and adult android fat mass (difference = 0.5 kg, 95% CI 0.1, 0.8, *p* = 0.006) (Figure 3F) compared with Q1 in men only. Similarly, rehabilitation weight gain in ΔWAZ/day (Q5 vs Q1) was significant in men. These relationships were not significant in women (Figure 3).

## 4 Discussion

In this cohort of adult Afro-Caribbean SM survivors, those who were less underweight at the time of hospitalisation for SM as children had greater adiposity as adults. Importantly, this positive association between minimum WAZ and adult adiposity was partially mediated by faster rehabilitation weight gain. Previous studies from this cohort suggest greater cardiometabolic risk in adult SM survivors compared to controls, viz, smaller outflow tracts and markedly elevated peripheral resistance (suggesting greater likelihood of developing excess hypertension) (Tennant et al. 2014) and distinct metabolic profiles (reduced β-oxidation and greater risk of type 2 diabetes) (Thompson et al. 2020). This study adds to our understanding of mechanisms that could explain greater cardiometabolic NCD risk in survivors of severe malnutrition.

Although in our data, the minimum weight-for age z score appears to have conflicting associations with weight gain expressed as g/day versus g/kg/day, we contend that whereas g/day measures the *absolute* daily change in weight (i.e., larger children at admission gain more weight daily on average), g/kg/day measures the daily rate of gain *relative* to body weight (i.e., larger babies at admission gain decreasing proportions of their weight every day).

A key finding of this study is the role that male sex plays in the relationship between rehabilitation weight gain and adult adiposity. Boys have been shown to grow faster than girls from an early stage of gestation, even from before implantation, and this makes them more vulnerable if their nutrition is compromised (Pedersen 1980). Among children born in Helsinki, boys tended to be longer than girls at any placental weight, suggesting that boy’s placentas are more efficient but may have less reserve capacity, which increases their vulnerability to undernutrition (Forsen et al. 1999). Furthermore, boys aged 0-59 months were shown to be much more likely to be wasted, stunted and underweight than girls of the same age using anthropometric case definitions, suggesting sex differences in susceptibility to undernutrition (Thurstans et al. 2020). One factor that potentially explains this finding is leptin, the satiety hormone, which is produced by white adipose tissue and tends to be higher in girls. Lower leptin levels trigger the production of ghrelin which increases the catabolism of the body’s energy stores (Klok et al. 2007).

In keeping with previously documented sex differences (Thurstans et al. 2022), we report that boys were more underweight, wasted, and stunted on admission for SM. Social, genetic, and environmental factors may affect sex differences in nutritional status, with the increase in risk being less pronounced for boys in South Asia, for example (Thurstans et al. 2022). However, in our study, despite boys being more underweight than girls at the time of admission to and discharge from hospital and despite gaining weight at the same rate as girls, those boys who experienced fastest weight gain had greater risk of adiposity as adults. In totality, our data suggest that boys may be more susceptible to the effects of nutritional rehabilitation if weight gain is too rapid. Adults who suffered from SM during childhood had less fat-free mass than an unexposed control group, but this observation was more marked in males than in females (Mwene-Batu et al. 2021), suggesting that males and females appear to be programmed differently for fat and lean mass accretion. Additionally, our finding of an association between faster rehabilitation weight gain and adult adiposity in male participants is in keeping with reports that NCD risk is greater in children who start out small and undergo rapid weight gain during critical windows [Singhal 2017 #1173].

While adjusting for sex in regression analyses is commonplace, it has the effect of simply removing sex from the analysis rather than considering sex as a variable of importance to the research question. Shapiro et al in their commentary urge that, instead of just controlling for sex, researchers should consider sex as a variable of importance that can explain, rather than confound, their research (Shapiro et al. 2021). Furthermore, there are well-established differences in body composition between adult men and premenopausal women with respect to fat mass, android fat and gynoid fat. In our regression models, the fact that the associations between minWAZ and adult adiposity outcomes are significant in sex-aggregated analyses, but in neither males nor females when sex is disaggregated, suggests that the combined model is likely reflecting the large effect of females having greater average fat mass than males.

By separating the sexes, we demonstrated that previously reported associations between adult adiposity and rehabilitation weight gain exceeding 12.9 g/kg/day or 0.09 WAZ/day (Thompson et al. 2023) are significant in men only and not in women. From a methodological perspective, this finding emphasises the importance of evaluating the role of sex in the pathophysiology of disease. This is a cautionary tale from which we should learn the greater value of disaggregating data by sex, to truly grasp its role with respect to the outcomes of interest.

Not surprisingly, we demonstrated that female sex is the strongest predictor of total fat mass in adult survivors. However, the findings of female sex being the strongest predictor of android fat as well as predicting waist circumference (albeit to a lesser extent) were unexpected. Sex differences in body composition are primarily due to the action of sex steroid hormones, which drive the dimorphisms during pubertal development. Age is also an expected important predictor, as fat mass is known to increase with age, with visceral fat deposition increasing as levels of oestrogen and testosterone fall (Seidell et al. 1990).

The fact that oedematous malnutrition (as opposed to severe wasting) was shown to be a predictor of adult adiposity is curious and warrants investigation. Our data suggest that this might not be a reflection of SM phenotype per se, as children with oedematous malnutrition were less under-weight on admission as well as at the time of minimum WAZ when all oedema was lost compared to children with severe wasting. However, as intermediary metabolism is more severely disrupted during oedematous malnutrition, where children break down fat, oxidise fatty acids less efficiently than do children with severe wasting (Badaloo et al. 2006) and suffer from hepatic steatosis, it is possible that children who had oedematous malnutrition could undergo a metabolic “re-set” that influences lipid metabolism and subsequent body composition in later life. The ‘fat overflow hypothesis’ posits that the capacity to increase the size and number of adipocytes is finite, and that when this limit is exceeded, fat accumulates in ectopic sites and leads to metabolic disease (Girousse et al. 2018). It would be instructive to further investigate this possibility by assessing lipid kinetics in adult survivors of severe wasting and oedematous malnutrition.

Our study demonstrated that children who were least underweight on admission for SM had greater adiposity as adults. We also showed that rehabilitation weight gain partially mediated the relationship between admission weight-for age and adiposity. Systematic reviews have consistently shown that rapid weight gain in infancy is associated with adiposity in later life (Zheng et al. 2018). What remains unclear is whether faster weight gain during treatment for SM is itself a direct contributor to adult adiposity, or whether it is simply a marker of an individual's capacity to accrete fat at any given stage. Given that only 13% of the effect of minimum WAZ on adult fat mass is mediated by rehabilitation weight gain (as g/kg/day), it is clear that other factors contribute to a greater extent to adult adiposity in this group. It is possible that exposure to SM or rapid weight gain during rehabilitation could individually or together re-set lipid kinetics resulting in greater adult adiposity. These questions could not be answered by this study and would be important areas of future research. We also acknowledge that many factors beyond SM and its treatment (adult diet, physical activity) may have a significant impact on adult adiposity and other cardiometabolic risk factors.

### 4.1 Strengths and Limitations

This study utilised a unique cohort of adult SM survivors with detailed anthropometric and body composition measures as children and adults. The low in-patient mortality rate (~ 4%) minimises the risk of survivor bias. Post-hospitalisation factors occurring in childhood, including the home diet and inter-current illnesses, could have influenced the observed associations and there could also be nonlinear aspects of growth that we were unable to capture. Additionally, data relating to adult diet and dietary intake (i.e., food insecure vs. obesogenic), physical activity, and co-morbid conditions, all of which may confound the observed associations, were not available for evaluation. Given the mixed SM phenotypes, it is likely that the study was underpowered to say something unique about oedematous malnutrition vs severe wasting or the independent effect of BW. Secondly, today’s treatments for SM are markedly different to those experienced by our cohort with the focus on outpatient care and slower average weight gain. (Bhutta et al. 2017). Community-based management of acute malnutrition (CMAM) programmes typically use a weight gain target of 4–5 g/kg/day. Our findings may therefore not apply or may differ in children affected by SM today. Finally, the participants in this study were Afro-Caribbean and the findings may be different in other ethnic groups.

## 5 Conclusions

In this group of Afro-Caribbean survivors of severe childhood malnutrition, faster rehabilitation weight gain mediated the association between the degree of underweight on admission and adult adiposity. Faster rates of weight gain during nutritional rehabilitation were shown to be a risk factor for adiposity in male adult SM survivors, suggesting a risk of worse outcomes in this specific group. These findings show that boys were not only more prone to becoming more severely malnourished than girls, but they were also more susceptible to the long-term effects of faster rehabilitation weight gain. Furthermore, as we demonstrated that many of the apparent associations with later life NCD are in fact the result of sex-based confounding, future analyses of this type should report sex-disaggregated data. To conclude, this study adds to the growing evidence of sex differences with respect to the risk and consequences of severe malnutrition and might suggest the need to take a more cautious approach to rehabilitation weight gain in general but particularly in males to mitigate the risk of cardiometabolic NCDs later in life.

## Supplementary Material

Supporting Information

## Figures and Tables

**Table 1:** Clinical characteristics of 278 adult survivors of severe malnutrition.

Participant characteristics	All participants (*n* = 278)	Men *(n* = 167)	Women (*n* = 111)
Birth weight (kg)	2.82 (0.78)^a^	2.90 (0.80)	2.70 (0.75)
Admission			
Age (days)	362 (173)	366 (191)	356 (143)
Oedematous malnutrition n (%)	182 (65.5)	111 (66.5)	71 (64.0)
Weight (kg)	5.43 (1.5)	5.38 (1.4)	5.51 (1.5)
Height/length (cm)	64.6 (7.0)	64.5 (7.1)	64.8 (7.0)
Weight-for-age z-score (WAZ)	–4.38 (1.5)	–4.72 (1.4)	–3.88 (1.4)**
Length/height-for-age z-score (HAZ)	–3.86 (1.9)	–4.28 (2.0)	–3.21 (1.6)**
Weight-for-length z-score (WHZ)	–3.11 (1.4)	–3.33 (1.4)	–2.82 (1.6)*
Rehabilitation phase (minimum weight to maximum weight)			
Change in weight-for age z-score (ΔWAZ/day)	0.07 (0.03)	0.07 (0.03)	0.07 (0.03)
Weight gain (g/d)	61.6 (25.3)	61.0 (26.2)	62.7 (24.0)
Weight gain (g/kg/d)	10.1 (3.8)	10.0 (3.6)	10.2 (3.9)
Duration of rehabilitation weight gain (days)	36.7 (19.0)	37.8 (21.1)	35.1 (15.2)
Discharge			
Weight-for-age z-score at discharge	–2.58 (1.45)	–2.87 (1.43)	–2.14 (1.36)**
Adult anthropometry and body composition			
Age (years)	26.5 (22.1, 33.4)	27.0 (22.2, 33.8)	26.0. (21.6, 33.4)
Weight (kg)	66.1 (14.9)	65.5 (11.7)	67.0 (18.7)
Height (cm)	167.5 (8.9)	171.1 (7.6)	162.0 (7.8)**
BMI (kg/m^2^)	23.6 (5.2)	22.3 (3.4)	25.5 (6.7)**
Waist circumference (cm)	78.8 (12.7)	76.3 (8.9)	82.6 (16.2)**
Fat mass (kg)	9.7 (4.3, 22)	5.1 (3.5, 9.8)	23.5 (12.3, 31.8)**
Fat mass index (kg/m^2^)	5.3 (5.0)	2.7 (2.5)	9.2 (5.3)**
Android fat mass (kg)	0.66 (0.27, 1.6)	0.36 (0.23, 0.73)	1.64 (0.79, 2.62)**
Lean mass (kg)	48.4 (10.4)	54.4 (7.5)	39.3 (14.1)**

*Note:* Student’s *t* test was used to compare the means between the two groups. Data are expressed as means (SD) or medians (25th percentile, 75th percentile).

a data for 152 participants (M-90, F-62); Significant difference between sexes.

* *p* < 0.01;

** *p* < 0.001. Obesity (BMI > 30 kg/m^2^): Total = 32, male = 6, female = 26.

**Table 2:** Sex-disaggregated linear regression analyses of the measures of adult adiposity against minimum weight for age in 272 adult SM survivors.

	Min WAZ as a predictor		All *n = 272*							Male *n* = 165							Female n = 107				
			B	(SE)	β	95%	CI	*p* value		B	SE	β	95%	CI	*p* value		B	SE	β	95% CI	*p* value
Waist	Model 1		1.78	0.55	0.19	0.70,	2.86	0.001		0.71	0.52	0.11	–0.32,	1.73	0.174		2.20	1.19	0.18	–0.15, 4.55	0.067
circumference (cm)	Model 2		1.37	0.56	0.15	0.27,	2.4	0.015		0.41	0.53	0.06	–0.64,	1.46	0.440		1.27	1.24	0.10	–1.19, 3.72	0.309
	Model 3		1.72	0.58	0.19	0.57,	2.87	0.003		0.69	0.56	0.10	–0.41,	1.80	0.218		1.66	1.31	0.13	–0.94, 4.27	0.208
	Model 4		1.98	0.53	0.22	0.94,	3.02	< 0.001		0.72	0.52	0.11	–0.31,	1.74	0.171		2.67	1.11	0.22	0.46, 4.88	0.018
Fat mass (kg)	Model 1		2.44	0.55	0.26	1.35,	3.53	< 0.001		0.32	0.43	0.06	–0.52,	1.16	0.459		1.85	1.03	0.17	–0.19, 3.89	0.075
	Model 2		2.22	0.57	0.23	1.09,	3.34	< 0.001		0.13	0.44	0.02	–0.73,	0.99	0.766		0.99	1.07	0.09	–1.13, 3.11	0.355
	Model 3		2.71	0.59	0.29	1.55,	3.86	< 0.001		0.48	0.46	0.09	–0.42,	1.38	0.295		1.25	1.13	0.12	–0.99, 3.49	0.271
	Model 4		2.86	0.57	0.31	1.74,	3.99	< 0.001		0.49	0.45	0.09	–0.39,	1.37	0.274		2.00	1.02	0.19	–0.02, 4.02	0.052
Android fat mass (kg)	Model 1		0.19	0.05	0.23	0.09,	0.29	< 0.001		0.03	0.04	0.07	–0.05,	0.11	0.406		0.14	0.10	0.13	–0.06, 0.33	0.165
	Model 2		0.17	0.05	0.21	0.07,	0.27	0.001		0.02)	0.04	0,04	–0.06,	0.10	0.748		0.07	0.10	0.07	–0.13, 0.28	0.486
	Model 3		0.21	0.05	0.25	0.10,	0.31	< 0.001		0.05	0.04	0.10	–0.04,	0.13	0.260		0.08	0.11	0.08	–0.14, 0.30	0.451
	Model 4		0.23	0.05	0.13	0.13,	0.33	< 0.001		0.05	0.04	0.13	–0.03,	0.13	0.230		0.16	0.10	0.15	–0.03, 0.35	0.09

Model 1: minWAZ Model 2: minWAZ + oedema Model 3: minWAZ + oedema + weight gain-g/kg/day quintiles* Model 4: minWAZ + oedema + weight gain-g/kg/day quintiles* + adult age

* Results were consistent when ΔWAZ was substituted for g/kg/day in models 3 and 4.

## Data Availability

Data for this manuscript can be accessed at London School of Hygiene and Tropical Medicine Data Compass Repository https://datacompass.lshtm.ac.uk/id/eprint/2656/.

## Summary

- Despite emerging evidence of an association between severe malnutrition (SM) in childhood and adult cardiometabolic noncommunicable disease risk, the factors that influence the association are unclear.
- Children who were older at the time of admission for SM experienced faster rehabilitation weight gain and those who weighed more at the time of admission for SM had greater adiposity as adults.
- Faster rehabilitation weight gain was an independent risk factor for greater adiposity (a proxy for cardiometabolic NCD risk) in adult male survivors only and rehabilitation weight gain partially mediated the positive association between minimum weight-for-age z scores and measures of adult adiposity
- The presence of nutritional oedema predicted adult adiposity.
- These findings add to the growing evidence of sex differences in the long- and short-term outcomes of severe malnutrition.
