# Supplementary material for: From Insult to Injury: Exploring the Associations Between Severe Malnutrition in Childhood, Rehabilitation Weight Gain and Adult Adiposity in a Prospective Cohort Study
Source: Matern Child Nutr. 2025 Sep 29;22(1):e70101. doi: 10.1111/mcn.70101 (PMC7618648; doi:10.1111/mcn.70101)
Supplement: Supplementary file 1 — Figure 1: Flow chart detailing recruitment of adult survivors of SM (n = 278). [file MCN-22-e70101-s001.docx]

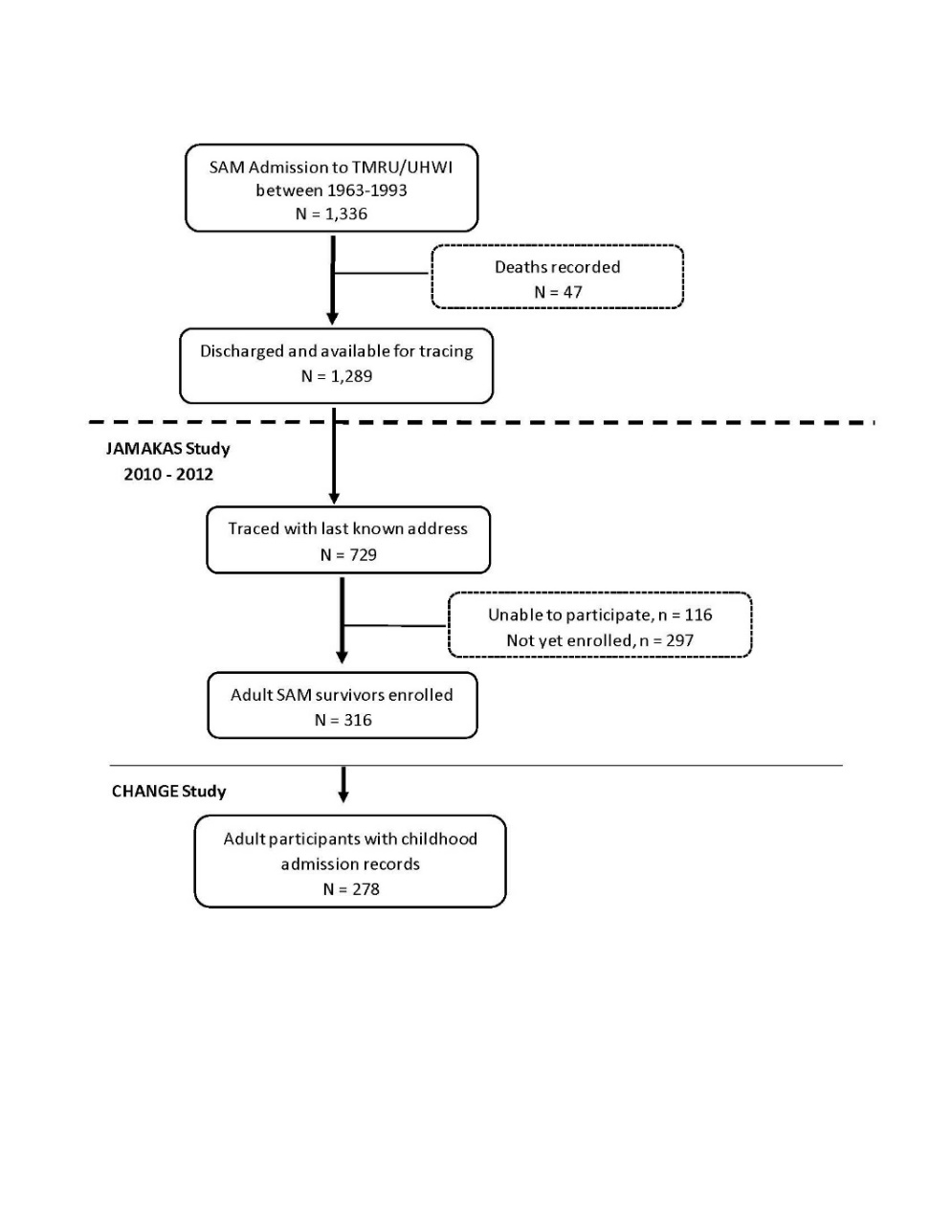


**Supplementary Figure 1**: Flow chart detailing recruitment of adult survivors of SM (*n* = 278). “Unable to participate” includes adult survivors of SM who were unavailable because of migration (*n* = 53), illness (*n* = 19), refusal (*n* = 14), or pregnancy (*n* = 30). TMRU, Tropical Metabolism Research Unit; UHWI, University Hospital of the West Indies; JAMAKAS, Jamaica Marasmus and Kwashiorkor Adult Survivors.
