# Supplementary material for: From Insult to Injury: Exploring the Associations Between Severe Malnutrition in Childhood, Rehabilitation Weight Gain and Adult Adiposity in a Prospective Cohort Study
Source: Matern Child Nutr. 2025 Sep 29;22(1):e70101. doi: 10.1111/mcn.70101 (PMC7618648; doi:10.1111/mcn.70101)
Supplement: Supplementary file 2 — Table 1: Summary of bivariate analyses of exposure variables (birthweight, oedema, minimum weight WAZ, sex, and rehabilitation weight gain expressed as quintile groups of WAZ/day, g/kg/day and g/day) against outcome variables (BMI, waist circumference, fat mass, % fat mass, android fat mass, % android fat and lean mass). [file MCN-22-e70101-s003.docx]

**Supplementary Table 1**: Summary of bivariate analyses of exposure variables (birthweight, oedema, minimum weight WAZ, sex, and rehabilitation weight gain expressed as quintile groups of WAZ/day, g/kg/day and g/day) against outcome variables (BMI, waist circumference, fat mass, % fat mass, android fat mass, % android fat and lean mass).

| **Dependent variable** | **Independent variable** | **All** | | | **Male** | | | **Female** | | |
| --- | --- | --- | --- | --- | --- | --- | --- | --- | --- | --- |
|  |  | **B coefficient (SE)** | **95% CI** | **p value** | **B coefficient (SE)** | **95% CI** | **p value** | **B coefficient (SE)** | **95% CI** | **p value** |
| BMI (kg/m^2^) | Birthweight | 0.12 (0.50) | -0.87, 1.1 | 0.812 | 0.33 (0.46) | -0.59, 1.3 | 0.478 | 0.16 (1.0) | -1.9, 2.2 | 0.879 |
|  | Oedema | 2.59 (0.65) | 1.33, 3.84 | **<0.001** | 1.6 (0.54) | 0.56, 2.7 | **0.003** | 4.2 (1.3) | 1.7, 6.7 | **0.001** |
|  | Min. WAZ | 0.62 (0.22) | 0.17, 1.06 | **0.007** | 0.07 (0.20) | -0.32, 0.46 | 0.732 | 0.77 (0.49) | -0.2, 1.7 | 0.120 |
|  | Min. WAZ age | <-0.01 (0.05) | -0.11, 0.11 | 0.989 | 0.02 (0.04) | -0.07, 0.10 | 0.715 | -0.02 (0.13) | -0.29, 0.25 | 0.893 |
|  | Sex-female | 3.15 (0.61) | 1.95, 4.35 | **<0.001** |  |  |  |  |  |  |
|  | WAZ/d | 0.42 (0.22) | -0.01, 0.85 | 0.053 | 0.42 (0.19) | 0.05, 0.79 | **0.027** | 0.23 (0.45) | -0.65, 1.11 | 0.610 |
|  | g/kg/d | 0.25 (0.22) | -0.18, 0.68 | 0.257 | 0.39 (0.19) | 0.02, 0.76 | **0.041** | -0.04 (0.45) | -0.92, 0.84 | 0.926 |
|  | g/d | 0.39 (0.22) | -0.04, 0.82 | 0.073 | 0.38 (0.19) | <0.01, 0.75 | **0.049** | 0.24 (0.44) | -0.63, 1.11 | 0.581 |
|  | Adult age | 0.19 (0.04) | 0.12, 0.27 | **<0.001** | 0.09 (0.03) | 0.03, 0.16 | **0.005** | 0.40 (0.08) | (0.24, 0.55 | **<0.001** |
| Waist circumference (cm) | Birthweight | 0.46 (1.2) | -1.9, 2.9 | 0.702 | 1.0 (1.2) | -1.4, 3.4 | 0.398 | 0.50 (2.4) | -4.4, 5.4 | 0.840 |
|  | Oedema | 5.7 (1.58) | 2.6, 8.8 | **<0.001** | 4.0 (1.4) | 1.1, 6.8 | **0.007** | 8.7 (3.1) | 2.5, 15 | **0.006** |
|  | Min. WAZ | 1.78 (0.55) | 0.7, 2.9 | **0.001** | 0.71 (0.52) | -0.32, 1.7 | 0.174 | 2.2 (1.2) | -0.15, 4.5 | 0.067 |
|  | Min. WAZ age | 0.12 (0.13) | -0.14, 0.39 | 0.353 | 0.17 (0.11) | -0.05, 0.39 | 0.124 | 0.04 (0.33) | -0.60, 0.69 | 0.896 |
|  | Sex- female | 6.3 (1.5) | 3.3, 9.3 | **<0.001** |  |  |  |  |  |  |
|  | WAZ/d | 1.19 (0.54) | 0.13, 2.24 | **0.028** | 1.13 (0.49) | 0.16, 2.11 | **0.023** | 0.86 (1.09) | -1.29, 3.01 | 0.429 |
|  | g/kg/d | 0.56 (0.54) | -0.51, 1.62 | 0.304 | 0.66 (0.50) | -0.33, 1.64 | 0.189 | 0.22 (1.09) | -1.93, 2.38 | 0.837 |
|  | g/d | 1.38 (0.54) | 0.32, 2.43 | **0.011** | 1.34 (0.50) | 0.36, 2.32 | **0.008** | 1.07 (1.07) | -1.05, 3.19 | 0.318 |
|  | Adult age | 0.61 (0.09) | 0.43, 0.80 | **<0.001** | 0.38 (0.08) | 0.22, 0.54 | **<0.001** | 1.08 (0.18) | 0.72, 1.44 | **<0.001** |
| Fat mass (kg) | Birthweight | -0.22 (1.2) | -2.6, 2.2 | 0.858 | 0.32 (1.0) | -1.7, 2.3 | 0.745 | 1.7 (2.1) | -2.6, 5.9 | 0.440 |
|  | Oedema | 4.2 (1.7) | 0.97, 7.5 | **0.011** | 2.3 (1.2) | -0.002, 4.7 | 0.050 | 7.9 (2.7) | 2.5, 13 | **0.005** |
|  | Min. WAZ | 2.4 (0.55) | 1.4, 3.5 | **<0.001** | 0.32 (0.43) | -0.52, 1.2 | 0.459 | 1.9 (1.0) | -0.19, 3.9 | 0.075 |
|  | Min. WAZ age | 0.02 (0.14) | -0.25, 0.29 | 0.903 | 0.11 (0.09) | -0.07, 0.29 | 0.236 | -0.12 (0.29) | -0.68, 0.45 | 0.681 |
|  | Sex-female | 16.3 (1.3) | 14, 19 | **<0.001** |  |  |  |  |  |  |
|  | WAZ/d | 1.17 (0.55) | 0.09, 2.26 | **0.033** | 1.01 (0.40) | 0.22, 1.80 | **0.013** | 0.27 (0.94) | -1.60, 2.13 | 0.776 |
|  | g/kg/d | 0.66 (0.55) | -0.43, 1.75 | 0.234 | 0.88 (0.40) | 0.08, 1.67 | **0.030** | -0.14 (0.94) | -1.99, 1.72 | 0.885 |
|  | g/d | 1.15 (0.55) | 0.06, 2.23 | **0.038** | 1.04 (0.40) | 0.25, 1.84 | **0.011** | 0.30 (0.93) | -1.54, 2.14 | 0.746 |
|  | Adult age | 0.34 (0.10) | 0.14, 0.54 | **0.001** | 0.16 (0.07) | 0.02, 0.30 | **0.023** | 0.78 (0.17) | 0.45, 1.12 | **<0.001** |
| Fat mass index (kg/m^2^) | Birthweight | -0.28 (0.47) | -1.2, 0.66 | 0.556 | 0.04 (0.35) | -0.65, 0.74 | 0.904 | 0.32 (0.83) | -1.3, 2.0 | 0.700 |
|  | Oedema | 1.5 (0.63) | 0.29, 2.8 | **0.016** | 0.76 (0.40) | -0.03, 1.5 | 0.061 | 3.0 (1.0) | 0.96, 5.0 | **0.004** |
|  | Min. WAZ | 0.86(0.21) | 0.44, 1.3 | **<0.001** | 0.07 (0.14) | -0.22, 0.35 | 0.651 | 0.50 (0.39) | -0.29, 1.3 | 0.212 |
|  | Min. WAZ age | 0.01 (0.05) | -0.10, 0.11 | 0.901 | 0.04 (0.03) | -0.03, 0.10 | 0.253 | -0.03, (0.11) | -0.24, 0.19 | 0.807 |
|  | Sex-female | 6.5 (0.48) | 5.6, 7.5 | **<0.001** |  |  |  |  |  |  |
|  | WAZ/d | 0.37 (0.21) | -0.04, 0.78 | 0.077 | 0.32 (0.14) | 0.06, 0.59 | **0.018** | -0.02 (0.35) | -0.73, 0.68 | 0.948 |
|  | g/kg/d | 0.19 (0.21) | -0.22, 0.61 | 0.359 | 0.30 (0.14) | 0.03, 0.57 | **0.029** | -0.16 (0.35) | -0.86, 0.55 | 0.659 |
|  | g/d | 0.34 (0.21) | -0.07, 0.75 | 0.107 | 0.33 (0.14) | 0.06, 0.60 | **0.018** | -0.04 (0.35) | -0.74, 0.65 | 0.899 |
|  | Adult age | 0.13 (0.04) | 0.05, 0.21 | **0.001** | 0.05 (0.02) | <0.01, 0.10 | **0.032** | 0.32 (0.06) | 0.20, 0.45 | **<0.001** |
| Android fat mass (kg) | Birthweight | -0.04 (0.11) | -0.26, 0.16 | 0.648 | 0.02 (0.09) | -0.16, 0.20 | 0.830 | 0.04 (0.19) | -0.35, 0.43 | 0.834 |
|  | Oedema | 0.33 (0.15) | 0.04, 0.62 | **0.025** | 0.18 (0.11) | -0.04, 0.40 | 0.101 | 0.61 (0.26) | 0.09, 1.1 | **0.022** |
|  | Min. WAZ | 0.19 (0.05) | 0.09, 0.29 | **<0.001** | 0.03 (0.04) | -0.05, 0.11 | 0.406 | 0.14 (0.10) | -0.06, 0.33 | 0.165 |
|  | Min. WAZ age | 0.01 (0.01) | -0.02, 0.03 | 0.632 | 0.01 (0.01) | <-0.01, 0.03 | 0.116 | -0.01 (0.03) | -0.06, 0.05 | 0.824 |
|  | Sex-female | 1.2 (0.12) | 1.0, 1.48 | **<0.001** |  |  |  |  |  |  |
|  | WAZ/d | 0.09 (0.05) | -0.01, 0.19 | 0.064 | 0.09 (0.04) | 0.02, 0.16 | **0.018** | 0.01 (0.09) | -0.17, 0.18 | 0.955 |
|  | g/kg/d | 0.05 (0.05) | -0.05, 0.14 | 0.358 | 0.07 (0.04) | <-0.01, 0.15 | 0.061 | -0.03 (0.09) | -0.21, 0.15 | 0.745 |
|  | 0.09 (0.05) | -0.01, 0.18 | 0.074 | 0.10 (0.04) | 0.02, 0.17 | **0.011** | <-0.01 (0.09) | -0.18, 0.18 | 0.988 | g/d |
|  | Adult age | 0.04 (0.01) | 0.02, 0.06 | <0.001 | 0.02 (0.01) | 0.01, 0.03 | **0.002** | 0.08 (0.02) | 0.05, 0.11 | **<0.001** |
| Lean mass (kg) | Birthweight | 3.2 (1.0) | 1.2, 5.2 | **0.002** | 2.4 (0.92) | 0.56, 4.2 | **0.011** | 1.4 (0.93) | -0.51, 3.2 | 0.150 |
|  | Oedema | 3.6 (1.3) | 1.0, 6.1 | **0.006** | 3.8 (1.2) | 1.4, 6.2 | **0.002** | 2.5 (1.4) | -0.18, 5.2 | 0.068 |
|  | Min. WAZ | -0.48 (0.46) | -1.4, 0.42 | 0.294 | 0.71 (0.44) | -0.16, 1.6 | 0.108 | 1.9 (0.48) | 0.92, 2.8 | **<0.001** |
|  | Min. WAZ age | -0.03 (0.11) | -0.25, 0.19 | 0.774 | -0.05 (0.09 | -0.24, 0.13 | 0.560 | -0.07 (0.14) | -0.35, 0.21 | 0.604 |
|  | Sex-female | -15 (0.89) | -17, -13 | **<0.001** |  |  |  |  |  |  |
|  | WAZ/d | 0.29 (0.45) | -0.59, 1.17 | 0.515 | 0.51 (0.42) | -0.32, 1.34 | 0.226 | 1.10 (0.46) | 0.19, 2.01 | **0.018** |
|  | g/kg/d | 0.11 (0.45) | -0.77, 0.98 | 0.813 | 0.16 (0.42) | -0.67, 0.99 | 0.706 | 0.49 (0.47) | -0.43, 1.42 | 0.296 |
|  | g/d | 0.47 (0.45) | -0.41, 1.35 | 0.294 | 0.45 (0.43) | -0.39, 1.29 | 0.296 | 1.47 (0.44) | 0.59, 2.35 | **0.001** |
|  | Adult age | 0.12 (0.08) | -0.04, 0.28 | 0.135 | 0.10 (0.07) | -0.05, 0.24 | 0.191 | 0.04 (0.09) | -0.14, 0.22 | 0.661 |

Birthweight: kg; Oedema: 1-yes, 0-no; MinWAZage: months; Sex: female-1, male-0; WAZ/d: ΔWAZ/day in quintiles (Q1-Q5); g/d: Δgrams/day in quintiles (Q1-Q5); g/kg/d: Δgrams/kg/day in quintiles (Q1-Q5); Adult age: years.
