# Supplementary material for: From Insult to Injury: Exploring the Associations Between Severe Malnutrition in Childhood, Rehabilitation Weight Gain and Adult Adiposity in a Prospective Cohort Study
Source: Matern Child Nutr. 2025 Sep 29;22(1):e70101. doi: 10.1111/mcn.70101 (PMC7618648; doi:10.1111/mcn.70101)
Supplement: Supplementary file 3 — Table 2: Predictors of adult adiposity in 147 adult survivors of childhood severe acute malnutrition. [file MCN-22-e70101-s002.docx]

**Supplementary** **Table 2:** Predictors of adult adiposity in 147 adult survivors of childhood severe acute malnutrition.

| **Dependent variables** | **Ranking of Predictive**  **Independent Variables** | **Independent**  **Variables** | **Unstandardized coefficient**  **B** | **SE** | **95%**  **CI** | ***p* value** |
| --- | --- | --- | --- | --- | --- | --- |
| **Waist circumference (cm)** | First | Adult age | 0.67 | 0.14 | 0.39, 0.94 | **<0.001** |
|  | Second | Oedema | 5.5 | 1.7 | 2.04, 8.9 | **0.002** |
|  | Third | Sex (female) | 3.6 | 1.7 | 0.17, 7.0 | **0.040** |
| **Fat mass (kg)** | First | Sex (female) | 12.7 | 1.5 | 9.6, 15.7 | **<0.001** |
|  | Second | Adult age | 0.38 | 1.2 | 0.14, 0.62 | **0.003** |
|  | Third | Oedema | 4.4 | 1.5 | 1.3, 7.4 | **0.005** |
| **Android fat mass (kg)** | First | Sex (female) | 0.91 | 0.14 | 0.63, 1.2 | **<0.001** |
|  | Second | Adult age | 0.04 | 0.01 | 0.02, 0.07 | **<0.001** |
|  | Third | Oedema | 0.33 | 0.14 | 0.06, 0.61 | **0.019** |

Covariates included were birth weight, WAZ at minimum weight (minWAZ), age at minimum WAZ (minWAZage), oedema (1-yes, 0-no), rehabilitation weight gain (as ΔWAZ/day, g/kg/day, g/day), sex (male - 0, female -1) and adult age.
